# Debonding Size Estimation in Reinforced Concrete Beams Using Guided Wave-Based Method

**DOI:** 10.3390/s20020389

**Published:** 2020-01-10

**Authors:** Beata Zima, Rafał Kędra

**Affiliations:** Department of Mechanics of Materials and Structures, Faculty of Civil and Environmental Engineering, Gdańsk University of Technology, Narutowicza 11/12, 80-233 Gdansk, Poland; rafal.kedra@pg.edu.pl

**Keywords:** nondestructive testing, guided wave, debonding, reinforced concrete, damage detection, embedded bar

## Abstract

The following paper presents the results of the theoretical and experimental analysis of the influence of debonding size on guided wave propagation in reinforced concrete beams. The main aim of the paper is a development of a novel, baseline-free method for determining the total area of debonding between steel rebar embedded in a concrete cover on the basis of the average wave velocity or the time of flight. The correctness of the developed relationships was verified during the experimental tests, which included propagation of guided waves in concrete beams with the varying debonding size, shape and location. The analysis of the collected results proved that guided waves can be efficiently used not only in the debonding detection, but also in an exact determining of its total area, which is extremely important in the context of the nondestructive assessment of the load capacity of the reinforced concrete structures. The undeniable advantage of the proposed method is that there are no requirements for any baseline signals collected for an undamaged structure. The paper comprises of the detailed step by step algorithm description and a discussion of both the advantages and disadvantages.

## 1. Introduction

Reinforced concrete (RC) is one of the most popular materials in the civil engineering field. Building structures of significant durability, long structural life and low maintenance costs must consider not only a correct project and high quality of the workmanship, but also fast and effective damage detection procedures. Damage detection methods dedicated for RC structures must allow for monitoring of the development of the internal damages characterized by a relative small size e.g., debonding between the concrete cover and the internal steel inserts. For this reason, the development of nondestructive methods has been of significant importance in the last decades. One of the approaches guaranteeing minimal impact on the structural integrity is the guided wave propagation method [1]. The high effectiveness of the application of guided waves in the diagnostics of RC structures was presented in many studies [2,3,4,5,6,7,8]. The significant attention was focused on the detection of delamination at the steel–concrete interface, because it is the one of the most dangerous and at the same time difficult to monitor types of damage due to its inaccessibility and invisibility. The need for fast debondings detection in reinforced concrete arises from the fact that the quality and the total area of the bonding connection determines the load capacity of the entire structure. When the debonding occurs, the reinforced concrete loses strength and durability, which results from the steel and concrete cooperation. Thus, debonding significantly jeopardizes the structural reliability and safety.

The feasibility study of the debonding detection using high and low frequency waves in RC beams with a varying debonding length was conducted by Na et al. [9]. The quality of bonding connection between steel tendons and the concrete was investigated by Beard et al. [10]. Wu et al. ([11,12]) presented comprehensive studies including theoretical, numerical and experimental investigations of debonding detection in reinforced concrete using built-in piezoelectric sensors. In the paper [13] they reported the results of the investigation of detection of the bond deterioration of steel–concrete adhesive connections during pull-out tests and defined a bond deterioration index (BDI) which was based on the amplitudes of signals received for free bar, pristine and damaged beams. Kim et al. [14] develop a new reference-free nondestructive technique of debonding detection in carbon fiber-reinforced polymer-strengthened (CFRP) RC structures by adopting the time reversal (TR) process to guided wave propagation. The TR technique in debonding detection in the concrete beams characterized by different bonding conditions in (CFRP)-retrofitted RC structures was also used by Li et al. [15]. Wang et al. [16] developed a concrete–steel interface spectral element characterizing interface conditions to investigate guided wave propagation along the rod embedded in a concrete cover. Sharma et al. [17] reported the results of the ultrasonic monitoring of debonding in RC structures, which developed as a result of corrosion processes. They observed increasing signal strength with an increasing debonding size. Li et al. [18] also showed that the energy attenuation for both low and high frequencies of ultrasonic waves increases with decreasing debonding extents. Zhu et al. [19] detected macro-cracks in RC beams by using piezoelectric transducers mounted on the reinforcing bars embedded in two concrete slabs with various bonding conditions. The description of wave propagation phenomenon with a special emphasis on wave modes’ conversions in the debonded steel bars embedded in a grout was presented by Zima et al. [20]. It was shown that the complexity of the propagation phenomenon strongly depends on the debonding location, while the average wave velocity depends only on its length. Yan et al. [21] employed an impedance analysis and wave propagation to identify the debondings of various thicknesses and sizes in steel ultra-high performance concrete (UHPC) composite slabs. Zhao et al. [22] applied the TR method to detect and localize the defects along the steel–concrete interface. They performed the defect images through the cross-sectional scanning. Ng et al. [23] showed the results of an application of nonlinear Rayleigh wave in the debonding detection in the CFRP-concrete structure. Their reference-free method was based on the generation of the second harmonic triggered because of the contact nonlinearity at the debonding damage. The Rayleigh wave scattering at the debonding between the fiber reinforced polymer and the concrete was studied experimentally and numerically by Mohseni and Ng [24]. The wave-based method of the debonding detection at the CFRP-concrete interface and the quantitative evaluation of the structural defects was also developed by Wang et al. [25]. The quality of the bonding condition between the concrete and a steel plate was assessed by Ke et al. [26] on the basis of the dispersion of the antisymmetric Lamb mode. The bond-slip failure in a steel–concrete–steel sandwich structure was monitored nondestructively using wave propagation by Yan et al. [27]. The partial least square regression (PLSR) technique was employed to detect the gaps in the steel–concrete composite structures by Giri et al. [28]. Their method provided the use of a minimal number of sensors attached to the internal surfaces of the tested structures. Most recently, Xu et al. [29] investigated the interface bond condition between steel tube and concrete in a concrete-filled steel tube-using surface wave measurement. Meanwhile, despite the significant effort of developing the diagnostic methods based on guided waves which allow for debonding detection, an exact estimation of the debonding area still remains an unsolved issue.

The results shown in the following paper are the continuation of the studies of debonding detection in RC structures [30,31]. The paper presents the results of theoretical and experimental investigations of guided wave propagation carried out on RC beams with artificially introduced debonding of varying sizes, shapes and locations. The main aim of the paper is the development of a nondestructive method of determining the exact area of contact zone between steel rods and concrete cover using time-domain signals collected at one end of the tested structure. The main novel elements are formulas relating average wave velocity or the time of flight (ToF) and the total debonding area, which have been derived and verified during experimental tests conducted on RC beams with single and multiple debondings.

## 2. Debonding Size Estimation Using Guided Waves—Theoretical Background

To analyze the influence of the total debonding area on wave velocity, in the first step the debonding length and circumferential extent impacts must be considered separately. The derivation of relationships between wave velocity and debonding dimensions (length and circumferential extent) presented in the following paragraph are based on previously conducted studies shown in [30,31]. Based on the theory, the novel relationship, applicable to detection of debonding of any size, shape and location is derived.

**Figure 1:**
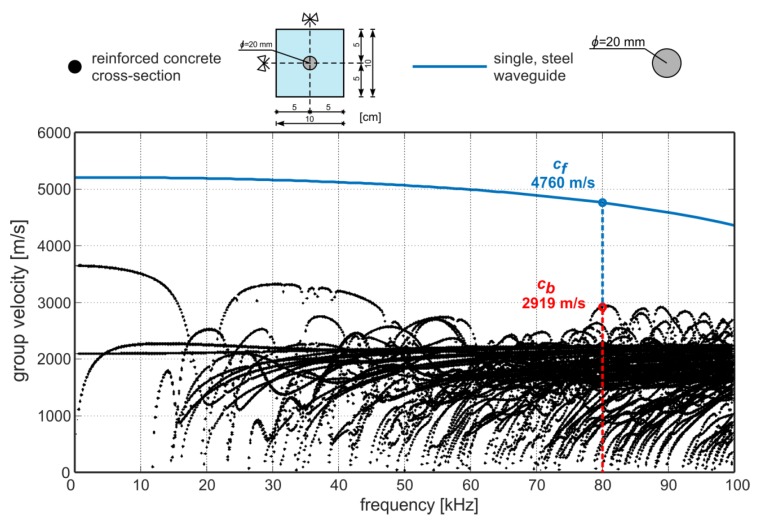
Dispersion curves for (•) reinforced concrete (RC) cross-section (concrete: *E* = 27 GPa, *v* = 0.18, *ρ* = 2290.4 kg/m^3^, steel: *E* = 212 GPa, *v* = 0.3, *ρ* = 7815 kg/m^3^), and for (-) uncovered, steel rod (*E* = 212 GPa, *v* = 0.3, *ρ* = 7815 kg/m^3^).

To describe in detail the wave propagation phenomenon and to derive the formulas relating debonding size with the average wave velocity, the solution of the dispersion equations need to be considered first. The solution is presented as dispersion curves, which relate the wave group velocity and the excitation frequency. The results presented in the previous papers ([30,31]) shown that, due to the gradual increase of debonding, the average wave velocity increases from *c_b_* (velocity in undamaged RC cross-section) to *c_f_* (wave velocity in free, steel bar). For this reason, when debonding damage is considered, two marginal cases need to be taken into account. The first case is a healthy, rectangular cross-section with a fully bonded and centrally embedded steel rod. The second marginal case is a totally debonded reinforcing steel rod. The theoretical solution in the form of dispersion curves for two different cross-sections considered in the further part of the paper is presented in Figure 1. Tracing the curves for the single rod requires solving the Pochhammer–Chree equation ([32,33]) which concerns only the longitudinal mode’s propagation. The Pochhammer–Chree equation was solved in the MATLAB software using our own code and the dispersion curve obtained is represented by a solid, blue line in Figure 1. Because during the experimental tests waves were excited only longitudinally, there is no need to consider the dispersion equations for other mode families (flexural and torsional).

**Figure 2:**
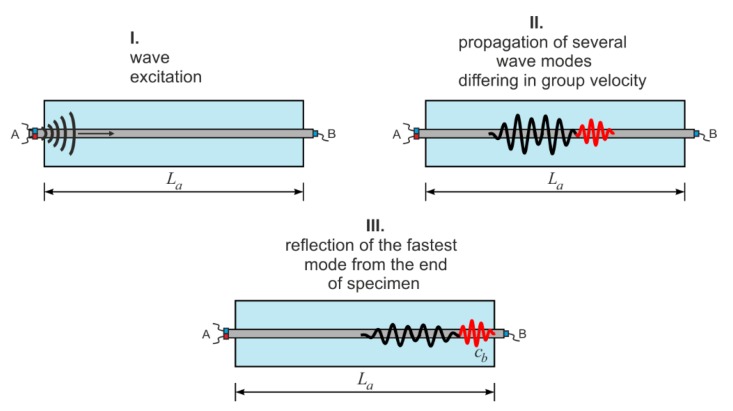
Multimode propagation in an undamaged RC beam.

Unfortunately, the complexity of the reinforced concrete cross-section excludes formulating the explicit dispersion equations. In this case, the dispersion curves were obtained using free software GUIGUW (Graphical User Interface for Guided Ultrasonic Waves) [34] based on the semi-analytical finite element method. The GUIGUW allows tracing of the dispersion curves for all wave mode families (longitudinal, flexural and torsional) and the type of mode represented by the individual curve can be recognized only on the basis of the associated wave structure. In general, it can be seen that the shape and the number of dispersion curves differs significantly for those two cases. In the case of the single, debonded waveguide, only one longitudinal mode can be excited, while for the RC cross-section the number of the possible solutions of dispersion equations is much larger, so it is even difficult to follow the individual curve. It means that wave excitation in a concrete beam, even for relatively low frequency (below 100 kHz), entails the multimode propagation. The schematic illustration of wave propagation in an undamaged concrete beam is given in Figure 2. After wave excitation, a number of modes start propagating (Figure 2—stage I and II). Not all modes are excited—when the wave is excited in the longitudinal direction, only symmetric modes are triggered. The mode with the highest velocity denoted as *c_b_* reaches the end of the beam first (Figure 2—stage III). The velocity *c_b_* in the RC beam for the considered excitation frequency can be determined on the basis of the dispersion solution (Figure 1). For example, in Figure 1 the theoretical velocity *c_b_* was determined for excitation frequency of 80 kHz. If the velocity *c_b_* is known, the ToF denoted as *t_a_* of the first reflection registered at the end *B* of the beam with total length *L_a_* can be then calculated as [30]:

(1) $$t_{a}^{}=\frac{L_{a}}{c_{b}}.$$

Modes propagating with the lower velocities are registered a signal later. When the reinforcing bar separates from the concrete cover, the continuity of the stress and displacement fields on the steel–concrete interface caused by the wave motion is disturbed and the average wave velocity cannot be determined on the basis of dispersion curves for the RC cross-section.

### 2.1. The Influence of Damage Length on Wave Velocity

**Figure 3:**
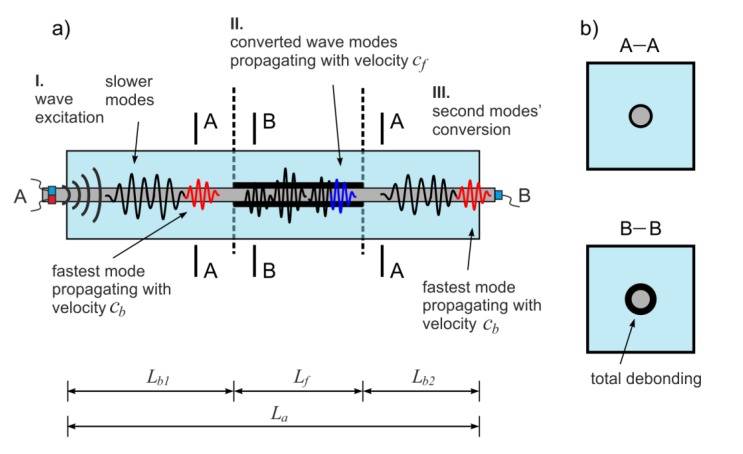
Wave propagation in RC beam with partial debonding damage: (**a**) Scheme of the phenomenon of wave modes’ conversion, (**b**) types of cross-sections.

The idea of the using waves in the debonding length determination is based on the wave modes’ conversion phenomenon. When in the specimen a multimode propagation is possible, the first reflection registered in a signal represents the fastest wave mode. Depending on whether the wave propagates in the debonded rod or in the healthy part of the beam, it travels with the velocity *c_f_* or *c_b_*, respectively (Figure 3). The velocity *c_f_* denotes velocity of the fastest mode in the free, debonded steel rod and as previously, it can be determined experimentally or theoretically on the basis of the dispersion curve (Figure 1). For the debonding length equal to *L_f_*, the formulas relating ToF and the average wave velocity *c_a_* with the debonding length *L_f_* and velocities of the fastest modes *c_f_* and *c_b_* can be derived:

(2) $$t_{a}^{}=\frac{L_{b1}}{c_{b}}+\frac{L_{f}}{c_{f}}+\frac{L_{b2}}{c_{b}}=\frac{L_{a}c_{f}-L_{f}\left(c_{f}-c_{b}\right)}{c_{b}c_{f}},$$

(3) $$c_{a}=\frac{L_{a}}{t_{a}}=\frac{L_{a}c_{b}c_{f}}{L_{a}c_{f}-L_{f}\left(c_{f}-c_{b}\right)},$$

where *L_b1_* and *L_b2_* denote the lengths of the bonded, undamaged parts of the specimen (Figure 3). The detailed description of wave propagation phenomenon in a partially debonded bar and the experimental verification of the above relationships are presented in [30]. It was shown that the total length of both single and multiple debondings can be determined on the basis of ToF measurements.

### 2.2. The Influence of Circumferential Debonding Size on Wave Velocity

The above reasoning presented in Section 2.1 concerned the case when only the length of the debonding *L_f_* varies, while the circumferential extent remains the same because the rod is totally debonded around the whole circumference (Figure 3b). In fact, the damage may develop gradually, which means that intermediate cases also have to be considered. The experimental results of the investigation of wave propagation in RC beams with debonding of a constant length but with a varying circumferential extent *d_e_* presented in [31] allowed for formulating of the relation between the velocity and the damage extent:

(4) $$c_{a}=\frac{2\pi r\cdot c_{b}c_{f}}{2\pi r\cdot c_{f}-d_{e}(c_{f}-c_{b})}.$$

**Figure 4:**
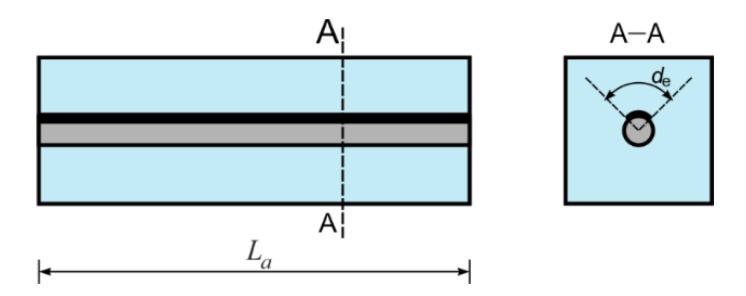
RC beam with circumferential debonding along the whole specimen length.

It can be seen that the average wave velocity *c_a_* does not depend on the total length of the specimen *L_a_* (Figure 4), because the theoretical considerations and the experimental tests were conducted only on the specimens with a rod debonded on the whole of its length and for constant values of *d_e_*. For this reason the Equation (4) is limited to such cases.

### 2.3. The Influence of Circumferential Debonding Size on Wave Velocity

**Figure 5:**
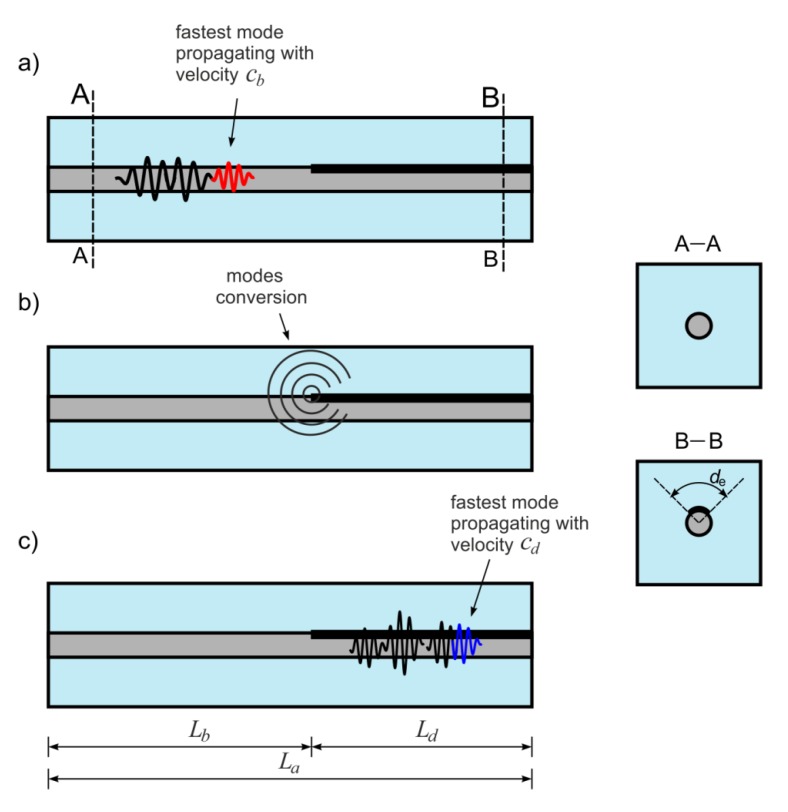
Wave propagation in RC beam with partial debonding: (**a**) wave propagation in undamaged part, (**b**) wave diffraction at the start of debonding, (**c**) wave propagation along debonded rod.

When the influence of debonding length and circumferential extent on the ToF is known, it is possible to derive the formula relating any debonding size with the ToF or the average wave velocity. Let’s consider the case of the RC beam with debonding of length *L_d_* and circumferential extent *d_e_* (Figure 5).

The velocity of the fastest mode in a healthy part, where the rod is fully bonded, is equal to *c_b_* (Figure 5a). After wave diffraction and modes’ conversion (Figure 5b), the velocity along the distance *L_d_* depending on the debonding extent is denoted as *c_d_* (Figure 5c) and it and can be calculated using Equation (4). Then, the average ToF can be calculated as:

(5) ta=Lbcb+Ldcd=Lbcb+Ld2πr·cbcf2πr·cf−de(cf−cb)=Lbcb+Ld(2πr·cf−de(cf−cb))2πr·cbcf=2πr·cf(La−Ld)2πr·cbcf++Ld(2πr·cf−de(cf−cb))2πr·cbcf=2πr·cfLa−2πr·cfLd+2πr·cfLd−Ldde(cf−cb)2πr·cbcf==2πr·cfLa−Ldde(cf−cb)2πr·cbcf

To obtain the more universal relationship that would take into account multiple debondings *n* with different lengths *L_d,i_* and circumferential extents *d_e,i_* the Equation (5) must be rewritten as:

(6) $$\begin{array}{l} t_{a}=\frac{L_{b}}{c_{b}}+\frac{L_{d,1}}{c_{d,1}}+\frac{L_{d,2}}{c_{d,2}}+\ldots +\frac{L_{d,n}}{c_{d,n}}=\frac{L_{b}}{c_{b}}+\sum_{i=1}^{n}\frac{L_{d,i}}{c_{d,i}}=\\ =\frac{2\pi r\cdot c_{f}\left(L_{a}-\sum_{i=1}^{n}L_{d,i}\right)}{2\pi r\cdot c_{b}c_{f}}+\sum_{i=1}^{n}\frac{L_{d,i}\left(2\pi r\cdot c_{f}-d_{e,i}(c_{f}-c_{b})\right)}{2\pi r\cdot c_{b}c_{f}}=\\ =\frac{2\pi r\cdot c_{f}L_{a}-\sum_{i=1}^{n}L_{d,i}d_{e,i}(c_{f}-c_{b})}{2\pi r\cdot c_{b}c_{f}} \end{array}$$

The velocity of the fastest mode can be obtained by dividing the total specimen length *L_a_* by the average ToF *t_a_*:

(7) $$c_{a}=\frac{L_{a}}{t_{a}}=\frac{L_{a}\cdot 2\pi r\cdot c_{b}c_{f}}{2\pi r\cdot c_{f}L_{a}-\sum_{i=1}^{n}L_{d,i}d_{e,i}(c_{f}-c_{b})}.$$

In Equation (7) the sum of the products of damage lengths and circumferential extents occurs, which is the total area of the debonding and is denoted as *A_d_*:

(8) $$A_{d}=\sum_{i=1}^{n}L_{d,i}d_{e,i}.$$

The Equation (7) can be rewritten in the following form:

(9) $$c_{a}=\frac{L_{a}\cdot 2\pi r\cdot c_{b}c_{f}}{2\pi r\cdot c_{f}L_{a}-A_{d}(c_{f}-c_{b})}.$$

Note that according to Equation (9), in the case of debonding damages with a variable length or circumferential extent, only their total area has an influence on the average wave velocity *c_a_*. It means that in two beams with different damages varying in shape, location etc. but with the same areas, waves would propagate with the same average velocity *c_a_*. Depending on the value of the difference $\left(c_{f}-c_{b}\right)$ occurring in the denominator, the average wave velocity may increase, decrease or remain the same for increasing debonding size *A_d_*. To determine the debonding area on the basis of the measured velocity, Equation (9) has to be reformulated:

(10) $$A_{d}=\frac{2\pi rL_{a}\cdot c_{f}\left(c_{a}-c_{b}\right)}{c_{a}\left(c_{f}-c_{b}\right)}.$$

The Equation (9) can be also presented in another form by dividing by $2\pi r\cdot L_{a}$:

(11) $$c_{a}=\frac{L_{a}\cdot 2\pi r\cdot c_{b}c_{f}}{2\pi r\cdot c_{f}L_{a}-A_{d}(c_{f}-c_{b})}|:\frac{L_{a}\cdot 2\pi r}{L_{a}\cdot 2\pi r}=\frac{c_{b}c_{f}}{c_{f}-\frac{A_{d}}{L_{a}\cdot 2\pi r}(c_{f}-c_{b})}=\frac{c_{b}c_{f}}{c_{f}-a_{r}(c_{f}-c_{b})},$$

where *a_r_* is a ratio between the area of the debonding damage *A_d_* and the total area of the side surface of embedded rod:

(12) $$a_{r}=\frac{A_{d}}{A_{rod}}=\frac{A_{d}}{2\pi r\cdot L_{a}}.$$

It can be seen that we can make the relative debonding size *a_r_* dependent on average velocity *c_a_* or ToF *t_a_*:

(13) $$a_{r}=\frac{c_{f}-\frac{c_{b}c_{f}}{c_{a}}}{c_{f}-c_{b}}=\frac{c_{f}c_{a}-c_{b}c_{f}}{c_{a}\left(c_{f}-c_{b}\right)}=\frac{c_{f}\left(c_{a}-c_{b}\right)}{c_{a}\left(c_{f}-c_{b}\right)},$$

(14) $$a_{r}=\frac{c_{f}\left(L_{a}-t_{a}c_{b}\right)}{L_{a}\left(c_{f}-c_{b}\right)}.$$

Note that the *a_r_* is linearly dependent on the ToF. If the ToF or average wave velocity *c_a_* is known, the relative debonding area can be easily estimated using the above equations.

## 3. Experimental Investigation

### 3.1. Description of Experimental Models

**Figure 6:**
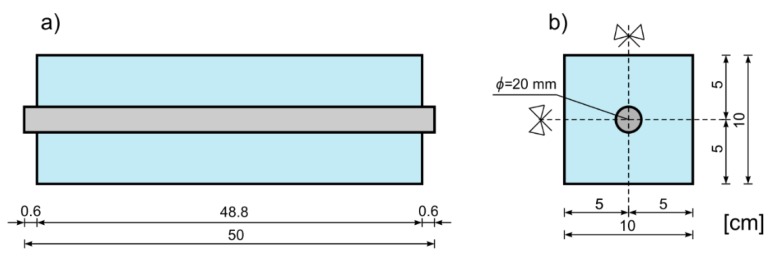
Geometry of the investigated beam: (**a**) Dimensions of the specimen, (**b**) geometry of the cross-section.

The correctness of the theoretical derivations presented in the previous chapter was tested experimentally. The nondestructive tests were conducted on RC beams with the rectangular cross section. The height of the beam was equal to 10 cm and the total length was 50 cm. The reinforcement in the form of one steel rod was placed longitudinally in the middle of concrete block. The radius of the rod was equal to *r* = 1 cm. The geometry of the specimen is shown in Figure 6.

The material parameters of the steel were determined by extensometric measurement during tensile tests and were as follows: Elastic modulus *E* = 212 GPa, Poisson’s ratio *v* = 0.3 and density *ρ* = 7815 kg/m^3^. The concrete mixture was made of Portland cement type 42 IIIR, sand (0–2 mm) and fine aggregates (2–8 mm). To determine the material parameters of the concrete, destructive tests were conducted on concrete samples with dimensions 150 mm × 150 mm × 150 mm. The parameters obtained for concrete were: Elastic modulus *E* = 27 GPa, Poisson’s ratio *v* = 0.18 and density *ρ* = 2290.4 kg/m^3^.

**Figure 7:**
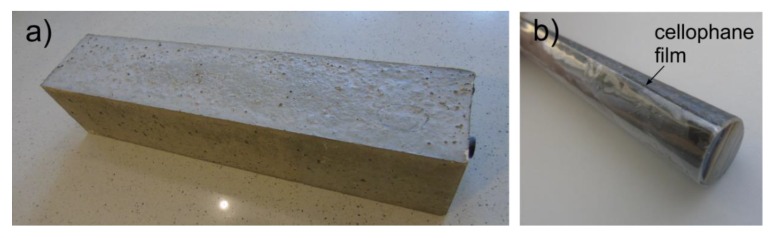
Experimental investigations: (**a**) Exemplary beam model, (**b**) rod covered with cellophane film before casting into concrete.

Six different damage scenarios were taken into account. The beams differed in the area of the debonding between the concrete cover and the steel bar. The debonding was simulated by placing a cellophane film between the rod and the cover, which provided a discontinuity of stress and displacement fields during wave propagation [35]. The photographs of the beam model and rod covered with the film before casting in concrete are shown in Figure 7.

**Figure 8:**
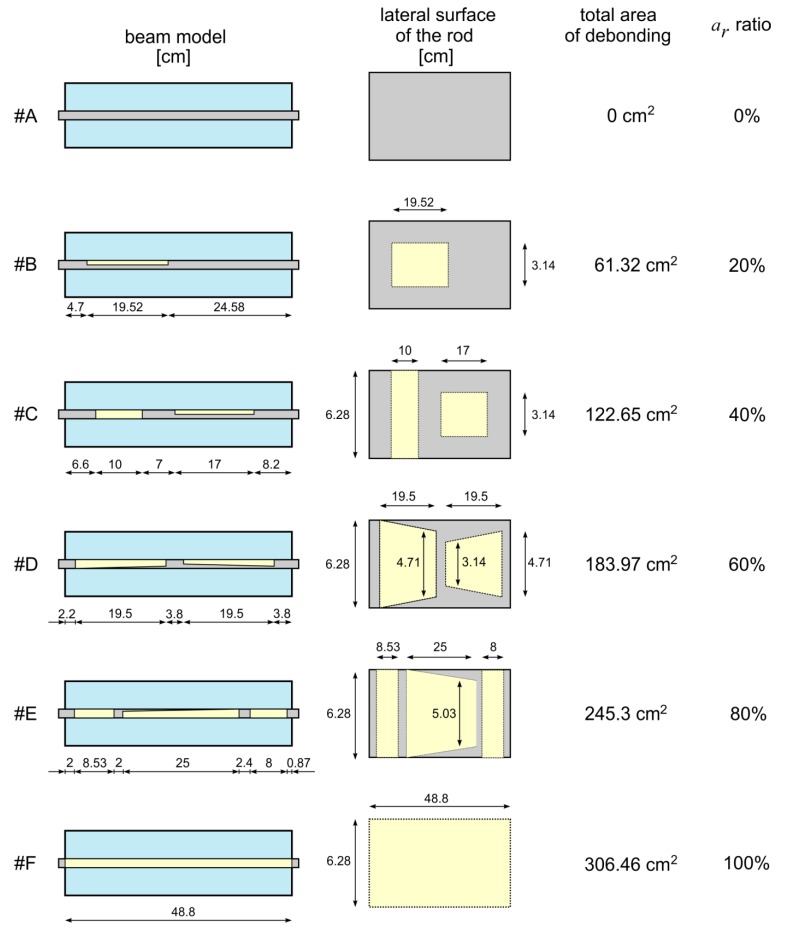
Damage scenarios considered in experimental tests: Beam with debonding of area equal to #A 0%, #B 20%, #C 40%, #D 60%, #E 80% and #F 100% of the total surface area of the rod embedded in concrete block.

All damage scenarios are summarized in Figure 8. The total surface area of the rod embedded in concrete beam was equal to:

(15) $$A_{rod}=2\pi rL_{a}.$$

For the rod used in the experiment (*r* = 1 cm, *L_a_* = 48.8 cm) the total surface area was 306.46 cm^2^. The relative debonding area *a_r_* was equal to 0%, 20%, 40%, 60%, 80% and 100%. The areas of debonding *A_d_* in centimeters squared were 0, 61.29, 122.58, 183.88, 245.17 and 306.46 cm^2^, respectively. The shape of the debonding and its location was chosen very freely-in the real concrete structures the debonding may develop in many places and take complicated, different shapes. The free choices of debonding position, length, circumferential extent, and whether damage was single or multiple, was to simulate the real, irregular damages occurring between rebars and a concrete cover. Moreover, the insignificant thickness of the film (90 μm) simulated the debonding at a very early stage of development.

Investigated beams were performed using smooth reinforcing rods, while actual reinforced concrete structures are reinforced with the ribbed bars to improve the quality of the steel and concrete grip. However, this work concerns the verification of novel relationships between the debonding area and wave velocity. For this reason, very high accuracy and meticulousness in the performance of the experimental models were required. The cellophane film adhered tightly to the rod, which prevented the entry of the cement slurry under its surface. In addition, a minimum amount of the adhesive was used only at the edges of the film so as to ensure slip between the film and the rod on the assumed area. Each film piece had specific dimensions that were selected so that the relative debonding area changed linearly for the subsequent beams. Meanwhile, the ribs would prevent the tight adhesion of the film to the rod, which could result in the penetration of cement slurry under the film surface. This in turn would prevent the accurate determination of the damaged concrete–steel connection area and, as a consequence, would significantly disturb the results obtained. Furthermore, in the case of non-smooth rods it would be much more difficult to introduce the artificial debonding with a complex shape that would cover an exact area of the ribbed rod (e.g., 80%). The last argument is that negligible ribs influence wave propagation velocity, which suggests that previously derived relationships and findings presented in the further parts of the paper are valid also for non-smooth bars [36].

### 3.2. Nondestructive Tests

**Figure 9:**
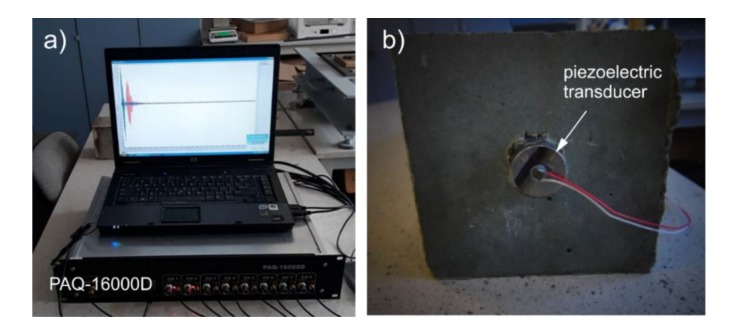
The photograph of (**a**) experimental equipment and (**b**) piezoelectric transducer attached to the end of the beam.

At the first stage, nondestructive tests were carried out. Guided waves were propagated and received with use of the PAQ-16000D device, and piezoelectric transducers Noliac NAC2012 attached at the both ends of the each beam (Figure 9). The input signal was in the form of a ten-cycle tone burst modulated by a Hann window. The results were collected for the carrier frequency of 80 kHz, however, the theoretical derivations presented in Section 2 are valid for any frequency. The frequency was chosen on the basis of a multi-criteria test. At first, the tuning test was made [37] and then the frequency from which the most readable signals with relatively high amplitude were registered was chosen. The tuning test was conducted only for the low frequency range 30–100 kHz, because in general, frequencies lower than 100 kHz should be utilized in damage detection in concrete structures to exclude the interactions of smaller wavelengths with the aggregates. The readability of the signal was the key factor of the frequency selection: We wanted the first package to be very clear, without any additional interfering packets to avoid the difficulties in results interpretation. Moreover, for a frequency of 80 kHz, there is quite a big difference between velocities *c_f_* and *c_b_* (see Figure 1), and the frequency of 80 kHz can be considered as sensitive to debonding detection. Despite that, the diagnostic procedure was carried out for excitation of 80 kHz, and the signals measured for other frequencies are presented in a further part of the work for comparison.

Next, the theoretical and experimental wave velocities in free rod *c_f_* and in RC beam *c_b_* were determined. On the basis of the dispersion curves the theoretical velocity in free rod $c_{f}^{t}$ was 4760.1 m/s and the theoretical velocity in undamaged beam $c_{b}^{t}$ was 2919 m/s. The experimentally determined wave velocities were as follows: $c_{f}^{e}$ = 4761.9 m/s and $c_{b}^{e}$ = 3472.2 m/s, and the difference between outcomes $c_{f}^{t}$ and $c_{f}^{e}$ for free, steel waveguide are insignificant, which proves the validity of the assumed theoretical dispersion model. However, in the case of velocities for the RC cross-section $c_{b}^{t}$ and $c_{b}^{e}$, the difference is noticeable. The first reason for this discrepancy is an assumption about perfect, isotropic, elastic and homogeneous materials. In the case of steel this assumption is fully justified, but in the case of the concrete, which is strongly inhomogeneous, this assumption could be not met. The complex microstructure of the concrete can lead to inaccuracies in the wave propagation model and also to wave scattering and some disturbing phenomena, e.g., faster wave attenuation. The second reason is imperfect geometry. Theoretical calculations were performed for rectangular cross-section with the steel rod casted perfectly centrally. The deviations from central position could cause velocity changes [38].

The third and the most influential reason is high sensitivity of velocity determination for deviations in the ToF measurements. There are a number of possible approaches for the ToF determination and the results obtained using these methods slightly differ from each other as shown by Xu et al. [39]. In this study only the simplest approach based on peak-to-peak value was involved. Moreover, experimental tests were conducted for relatively short specimens (0.5 m). For such a short beam even a very slight difference in ToF equal to 10^−5^ s resulted in an over- or underestimation of propagation velocity by several hundreds of m/s. All these aspects should be taken into account in the case of practical application of the proposed algorithm of debonding detection and its size estimation.

**Figure 10:**
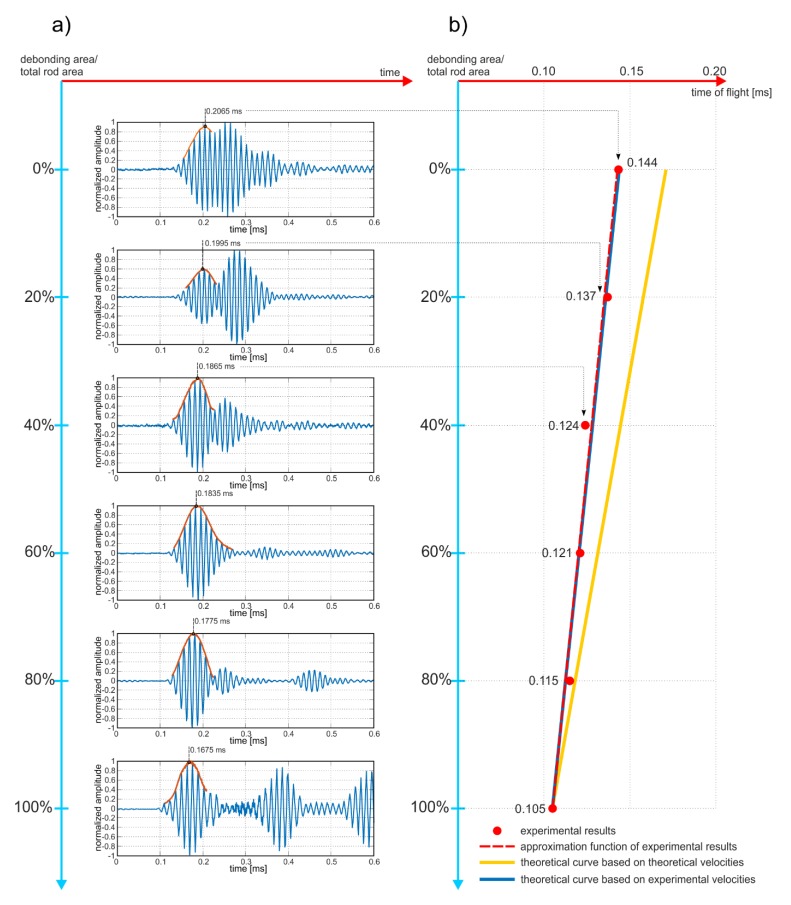
Results of experimental investigations: (**a**) Time-domain signals registered at the end of the beam, (**b**) values of the ToF for varying debonding areas.

Guided waves were propagated in beams from #A to #F (Figure 8). The results of the experimental investigations were collected in the form of time-domain signals registered at the end of the beam (Figure 10a). To calculate the ToF of the reflections, the envelopes of the signals were performed using the Hilbert transform. For each signal the ToF of the first peak was determined and indicated in the figure. It can be clearly seen that the travel time decreases with increasing size of debonding area, which is in high agreement with theoretical considerations (compare Equation (9)). To obtain the peak-to-peak value, the ToFs were reduced by a half the duration of the input wave packet (for excitation frequency of 80 kHz, half the time duration of the input packet was equal to 0.0625 ms), then were collected in the graph in Figure 10b and approximated by a linear function using regression analysis and the least squares method. The quality of the linear approximation is very high, as evidenced by the value of the coefficient of the determination *R*^2^ (0.977). To compare the experimental results and theoretical predictions, two additional functions were plotted. The first one, represented by a solid, blue line, was traced with the use of Equation (6) and the experimentally determined velocities $c_{f}^{e}$ and $c_{b}^{e}$. One can see that the approximation function and the theoretical solution perfectly coincide with each other, which proves the correctness of the proposed formula relating the ToF and the relative debonding area *a_r_*. The second function, represented by a solid, yellow line, was determined with the use of Equation (6) and theoretically determined velocities $c_{f}^{t}$ and $c_{b}^{t}$. In this case the difference between theoretical and experimental results is visible. The most significant discrepancy was obtained for the damage-free beam #A. The discrepancy between the theoretical and experimental results decreased with the increase of the debonding area until the ToF for the fully-debonded beam #E coincided perfectly. A varying discrepancy stems from differences in experimentally and theoretically obtained wave velocities. Velocities $c_{f}^{e}$ and $c_{f}^{t}$ are almost the same, while $c_{b}^{e}$ and $c_{b}^{t}$ differ by around 500 m/s. The smaller the debonding area, the more the average wave velocity *c_a_* (and in consequence the average ToF) tended to velocity $c_{b}^{e}$, and the more the deviation from the theoretical result $c_{b}^{t}$ was observable.

Regardless of the results deviations, all three functions clearly illustrate the linear decrease of the ToF with the increasing debonding area, which is consistent with Equation (14). This means that the travel time can be undoubtedly used as a qualitative parameter allowing for constant monitoring of the bonding condition. Any registered changes in the ToF may indicate the deterioration of adhesive connection quality.

**Figure 11:**
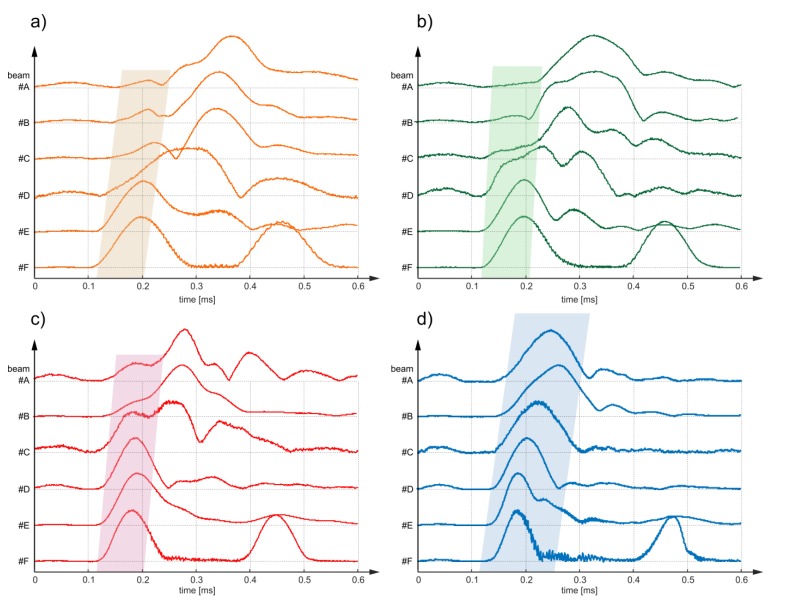
Results of experimental investigations in the form of envelopes of time-domain signals registered at the of beam for excitation frequency equal to (**a**) 50 kHz, (**b**) 60 kHz, (**c**) 70 kHz and (**d**) 90 kHz.

Despite the fact that the experimental analysis was conducted for a frequency of 80 kHz, the results for other frequencies are also presented for comparison. Figure 11 contains the sets of the signals’ envelopes for excitation frequencies: 50, 60, 70 and 90 kHz. The first reflections were identified and highlighted. In each case the ToF behaves linearly and decreases with an increase of debonding size, but the time-debonding area dependency is different for various frequencies.

Despite the downward trend in the registered ToFs being visible, the quality and thus the difficulty in interpreting the results also depends on the excitation frequency. Note that in some cases (e.g., Figure 11b—signal for the #A and #B beam), the peak envelope amplitude of the first wave measured is very difficult to identify due to its low energy, as well as interference from another high-energy reflection. The problem is particularly pronounced for the lower frequencies for which wave packets are relatively long. It can be seen that for higher frequencies (80 and 90 kHz) individual reflections are easily distinguishable. On the one hand, the use of high frequencies can improve the quality and legibility of the signal, but on the other hand, the higher the frequency, the higher the probability of dispersion, multimode propagation and interaction of the shorter wavelength with the aggregates, which also hinders signals interpretation.

## 4. Proposed Algorithm of Debonding Area Estimation

In this section we propose the procedure of debonding area estimation with particular emphasis on possible difficulties that may occur. On the basis of the obtained experimental signals, the relative debonding areas in experimental beams models are determined and compared with the exact ones. The algorithm of debonding size estimation is limited to three steps:

- The first step is determining the material and geometric parameters of the monitored structure and calculating the dispersion curves for the reinforced steel bar and RC cross-section. The curves are indispensable to determine the velocities *c_f_* and *c_b_*. If possible, the velocities can be also determined experimentally, which eliminates the necessity of curves’ trace calculations. In the presented study the theoretical velocities $c_{f}^{t}$ and $c_{b}^{t}$ were 4760.1 m/s and 2919 m/s and experimental velocities $c_{f}^{e}$ and $c_{b}^{e}$ were 4761.9 m/s and 3472.2 m/s, respectively (compare Section 3.2).
- The second step is an identification of the first arriving waveform, determining its ToF and calculating the average velocity *c_a_* in the investigated beam. In the experimental tests the wave velocities in particular beams for the excitation frequency of 80 kHz were as follows (Table 1):
- The last step is the calculation of the total debonding area *A_d_* or the relative debonding area *a_r_* with the use of Equations (10) or (13). On the basis of the experimentally determined velocities in the previous step, the ratio *a_r_* has been established for each beam and compared with the exact debonding area (Table 2). The calculations were performed using two different data sets. In Equation (13) experimental velocities $c_{f}^{e}$ and $c_{b}^{e}$ were substituted and the ratios $a_{r}^{e}$ were calculated. The results are presented in the third column in Table 2. Next column contains absolute errors calculated using the formula:
  (16) $$\delta_{e}=\left|a_{r}-a_{r}^{e}\right|.$$

The maximum value of the absolute error *δ_e_* does not exceed 11.3%, which is a highly satisfactory result. In the rest of the cases the error did not exceed several percentages (0–5.7%).

Further, in Equation (13) theoretical velocities $c_{f}^{t}$ and $c_{b}^{t}$ were substituted and the relative debonding areas $a_{r}^{t}$ determined are summarized in the fifth column. In this case, the differences between calculated and exact values of *a_r_* denoted as *δ_t_* are more significant. As expected, the highest value of the absolute error of 41% has been obtained for the damage-free beam #A (compare with Figure 10b). As mentioned in Section 3.2, this discrepancy results from the differences in theoretically and experimentally determined wave velocities in the undamaged beam. For beams with greater debonding areas the results obtained are much closer to exact values.

The results presented indicate the high potential of guided wave propagation in assessment of the exact debonding size, which is an indispensable prerequisite for the nondestructive determination of load capacity of RC structures. The presented algorithm is baseline-free, which is the main advantage of this approach. The next advantage is the simplicity of the entire diagnostic procedure. There is no need to use a wide area network of permanently attached sensors. The signal measurement can be made using a single sensor-actuator transducer pair installed at one point at the end of monitored structure. Moreover, only a basic parameter, which is the ToF of the first arrival wave packet, is monitored during the diagnostic process, and no complex signal processing tools are essential.

However, as is the case of any ToF-based method, the main disadvantage is its sensitivity to inaccuracies in the calculations of the ToF or wave velocities. The difference between theoretical and experimental velocities $c_{b}^{e}$ and $c_{b}^{t}$ led to an overestimation of the debonding size. From a practical point of view this problem can be solved by performing multiple measurements for several different excitation frequencies and various wave velocities $c_{f}$ and $c_{b}$. In this study for each beam the debonding area has been determined on the basis of the single signal. Collecting a greater number of signals for various parameters of the input wave packet allows for improvement of the accuracy of debonding size estimation.

## 5. Discussion of Aspects of Practical Application

Before the guided wave-based method of debonding detection can be applied in practice, it is necessary to consider the limitations of the presented approach. These limitations discussed below are also the directions for further research:

- Tracing the dispersion curves. Tracing the dispersion curves requires knowledge regarding the material parameters of steel and concrete. In the case of the investigation of the real object it is usually possible to establish their values on the basis of the design documentation. Otherwise, additional experimental destructive or nondestructive tests (e.g., conducting ultrasonic pulse velocity tests) on specially prepared samples removed from the structure, e.g., by core drilling, would be necessary.
- Excitation of the longitudinal modes. In the experimental research, waves were excited only longitudinally in the middle of the beam cross-section. For this reason, all theoretical reasoning presented in Section 2 was conducted for longitudinal modes (see Figure 1). In fact, it is not always possible to uncover the rod to attach the sensor and actuator to excite waves along its axis. The solution might be to use embedded transducers, however, this approach has serious practical limitations. From the practical point of view, the preferred solution would be attaching the transducers at the bottom or top beam surface and exciting waves perpendicularly to the specimen axis. In such a case flexural modes are also excited in the specimen. Their propagation must be taken into account during determination of the velocities of the fastest modes.
- Complex shape of the reinforcing bars. The bars investigated were smooth and straight, meanwhile complex geometry of the bars as well as the presence of additional reinforcement, such as stirrups, triggers additional wave reflections, and wave modes’ conversions, which undoubtedly affect the registered signals. Thus, the application of guided waves in damage detection in RC structures requires detailed recognition of the propagation phenomenon in specimens characterized by the complex geometry.
- Significant size of the investigated specimen. The experimental research has been performed on beams 50 cm long. Reinforced concrete beams are usually characterized by a greater length, which entails stronger wave attenuation. The debonding detection in actual engineering structures involves the necessity of generating high-energy excitation. In this study we used a single transducer with a free stroke of 3.3 µm and blocking force of 378 N to actuate the guided waves. The input signal driving the actuator was characterized by the amplitude not exceeding 10 V, while the maximum voltage for this transducer was 150 V. It means that for the same hardware configuration, the input signal can be multiplied by 10–15 times. Additionally, if necessary, a single actuator can be replaced by a piezoelectric stack, which allows for an additional increase of input wave amplitude up to 10 times. With the current technical parameters of piezo-actuators, testing of longer objects is possible with adequate amplifying of the excitation. Moreover, the analysis can also include the study of attenuation curves, which illustrate the intensity of wave damping depending on the excitation frequency. On the basis of the attenuation curves, the preferable frequency ensuring the low wave attenuation can be chosen.

## 6. Conclusions

The paper presents a debonding detection technique, which can be applied in diagnostics of RC structures. The developed method allows for exact debonding size determination on the basis of the single measurements made on the one end of the tested specimen. The novel relationships describing the influence of the total debonding size on average wave velocity and the time of flight have been derived. It was shown that there is a linear dependence between debonding size and travel time of the fastest wave mode. Experimental investigations conducted on a number of RC beams with varying debonding sizes, shapes and locations confirmed the correctness of the formulated relationships. It was shown that there is a linear dependence between debonding size and travel time of the fastest wave mode. A aigh agreement of results (a maximum percentage error of 11.29%) was obtained when calculations were based on experimentally determined wave velocities $c_{f}^{e}$ and $c_{b}^{e}$. In the case when wave velocities $c_{f}^{t}$ and $c_{b}^{t}$ were determined theoretically with the use of dispersion curves, the discrepancies between exact and calculated debonding areas were higher, but still the increase of average wave velocity with increasing debonding size was clearly visible. However, despite the promising results, the method has some difficulties that must be solved before practical application in the diagnosis of real reinforced concrete structures. Additionally, there are the technical aspects of the method utilization, which must be solved before practical application can be discussed.

## Figures and Tables

**Table 1:** Experimentally obtained average velocities *c_a_* in beams with varying debonding area.

Beam	Ratio *a_r_* [%]	Experimental Average Wave Velocity *c_a_* [m/s]
#A	0%	3472.2
#B	20%	3649.6
#C	40%	4032.3
#D	60%	4132.2
#E	80%	4347.8
#F	100%	4761.9

**Table 2:** Calculation of debonding area using theoretical and experimental results.

Beam	Relative Debonding Area *a_r_* [%]	Debonding Area $a_{r}^{e}$ Calculated with Use of Experimental Velocities	Percent Error *δ_e_*	Debonding Area $a_{r}^{t}$ Calculated with Use of Theoretical Velocities	Percent Error *δ_t_*
#A	0%	0	0%	41%	**41%**
#B	20%	17.94%	2.06%	51.75%	31.75%
#C	40%	51.29%	**11.29%**	71.4%	31.4%
#D	60%	58.97%	1.03%	75.9%	15.9%
#E	80%	74.36%	5.64%	84.97%	4.97%
#F	100%	100%	0%	100%	0%
